# Feminist contributions on sexual experiences of women with serious mental illness: a literature review

**DOI:** 10.1007/s00737-022-01258-0

**Published:** 2022-08-22

**Authors:** Kira Grachev, Valeria Santoro Lamelas, Anne-Sophie Gresle, Leonardo de la Torre, Maria-Jesus Pinazo, on behalf of the InSPIRES Consortium

**Affiliations:** 1 Barcelona Institute for Global Health (ISGlobal), Hospital Clínic-Universitat de Barcelona, 08036 Barcelona, Spain; 2 Interaction and Social Change Research Group (GRICS), Department of Social Psychology and Quantitative Psychology, Universitat de Barcelona, 08035 Barcelona, Spain

**Keywords:** Sexuality, Feminism, Serious mental illness, Women’s mental health

## Abstract

This paper aims to explore the contributions of research that include gender perspective in analysing the sexual experiences of women diagnosed with serious mental illness and to identify any barriers and systems that impede sexual fulfilment. We have developed a qualitative literature review using the PRISMA statement. The databases SCOPUS, WOS and PsychINFO were used in this review. Studies were included if they were published up to March 15, 2022, and only studies in English were included. An initial database search was preformed; upon screening for eligibility, there remained 16 studies that explored the sexual experiences of women with diagnoses of serious mental illness. The studies were analysed by a thematic synthesis. Data was coded line-by-line which generated descriptive themes, resulting in four synthesised findings. The four synthesised findings that derived from the reviewed studies were stigma and subjectivity, the experience of interpersonal relationships, the socialisation of women and the effects of psychiatric hegemony. A feminist perspective highlights the interrelationship between gender and stigma as it relates to serious mental illness and sexuality. A feminist perspective and an intersectional approach should be adopted at the intersubjective and structural level to account for the complexity of human experience and to subvert the heteropatriarchal system.

**Supplementary Information:**

The online version contains supplementary material available at 10.1007/s00737-022-01258-0.

## Background

Sexual rights are critical conditions for the fulfilment of sexuality (Parker 2007). Its exercise is indispensable in guaranteeing sexual health; understood as “a positive and respectful approach to sexuality and sexual relationships, as well as the possibility of having pleasurable and safe sexual experiences, free of coercion, discrimination and violence” (WHO 2006, p.5). Sexual rights determine an individual’s ability to choose; whether it be choosing to be sexually active or not or deciding if and when to have children. The realisation of such rights requires a comprehensive societal and institutional understanding of them, including an acceptance of the diversity of needs and experiences of individuals (Parker 2007).

Sexuality is an integral component of human identity and activity (Brown 2015). Sexuality is defined by the World Health Organization (WHO) as: “A central aspect of being human throughout life encompasses sex, gender identities and roles, sexual orientation, eroticism, pleasure, intimacy and reproduction. Sexuality is experienced and expressed in thoughts, fantasies, desires, beliefs, attitudes, values, behaviours, practices, roles and relationships. (….) Sexuality is influenced by the interaction of biological, psychological, social, economic, political, cultural, legal, historical, religious and spiritual factors” (WHO 2006, p. 5). Despite its complexities, research on sexuality has historically focused on potential risks to health, such as sexually transmitted diseases, and undesired pregnancies (Anderson 2013). Sexuality is increasingly being understood as more than the absence of negative or unwanted health outcomes (Anderson 2013). This current definition is a more comprehensive approach and understanding to sexuality and also recognises the significance and power that exists within the experience of sexuality, both individually and societally.

Sexuality and sexual rights are experienced differently among different populations for a variety of reasons. People with serious mental illness are particularly affected as they tend to face greater psychological, emotional, social and economic challenges than the rest of the population (Carr et al. 2015). This impacts their capacity to build relationships and self-esteem, both critical for sexual expression (Carr et al. 2015). Serious mental illness is defined as, “a mental, behavioural, or emotional disorder resulting in serious functional impairment, which substantially interferes with or limits one or more major life activities” (National Institute for Mental Health 2021). The societal stigmatisation of people with diagnoses of serious mental illness (DSMI) can considerably impact an individual’s subjective experience and can result in internalised stigma (Chronister et al. 2013). Given its interconnectedness with other parts of an individual’s identity, sexuality can also be affected by an individual’s experience of stigma.

Sexuality is an inherently gendered experience and achievement of sexual rights and free expression of sexuality require an analysis of gender. Sexuality and sexual orientation are inextricable from the social construction of gender, for example, classifications of heterosexuality and homosexuality are contingent on the social construct and differentiation of gender. The inclusion of a gender analysis allows for a better understanding of how the heteropatriarchal system impacts on the subjectivity construction in women and thus, regulates sexual practices.

The aim of this research was to conduct a literature review on studies that analysed the sexual experiences of women with DSMI and to synthesise the existing barriers and systems that may have impede the sexual experiences and sexual fulfilment of this population.

## Methodology

**Fig. 1:**
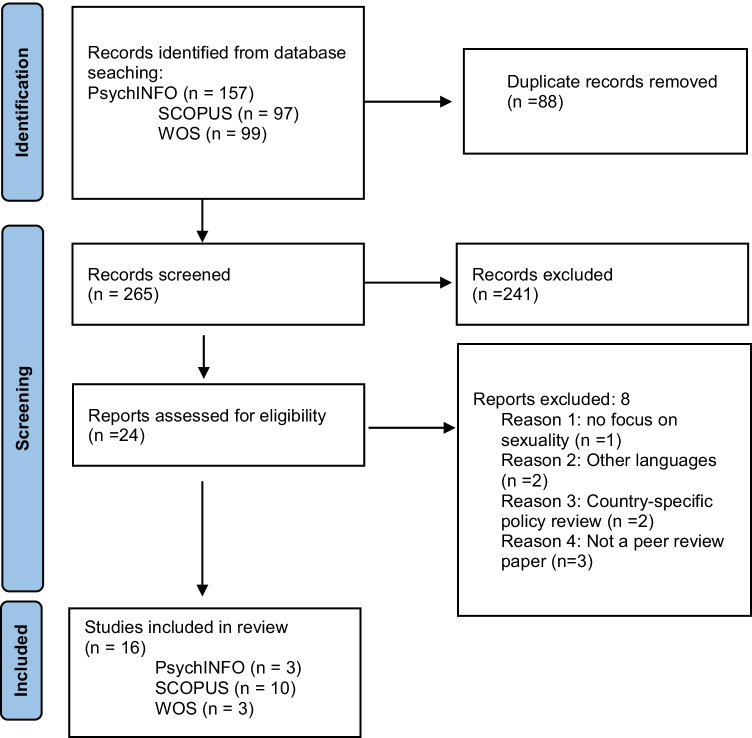
PRISMA flowchart of the search selection. *PRISMA Flowchart adapted from:* Page MJ, McKenzie JE, Bossuyt PM, Boutron I, Hoffmann TC, Mulrow CD, et al. The PRISMA 2020 statement: an updated guideline for reporting systematic reviews. BMJ 2021;372:n71. https://doi.org/10.1136/bmj.n71

This qualitative literature review followed the PRISMA guidelines, which provide guidance for rigorous, comprehensive and transparent systematic reviews (Page et al. 2021a, b). It includes a description of a series of steps and a checklist for searching, selecting and presenting studies in a literature review (Liberati et al. 2009). The PRISMA statement serves as a tool to guide the systematic review as well as to describe the process undertaken (Fig.1). The literature search was conducted from the SCOPUS, Web of Science (WOS) and PsychINFO (Ovid) databases; these databases are widely used sources of peer-reviewed high-impact publications. The last search occurred in March 2022, and the last articles were retrieved on March 15, 2022.

### Defining concepts

A variation of the following key concepts were searched in the subsequent order: *Sexuality AND “Serious Mental Illness” AND Feminism.*

For the purpose of this database search, sexuality was defined by an individual’s capacity to fulfil their sexual expression and practices. This includes their possibility to express their sexual orientation, to establish sexual and intimate relationships and to have autonomy over their sexual practices, including when and if they want to have sex and if and what kind of contraception is used.

*The second concept sought to identify articles that focused on populations living with serious mental illness. The Diagnostic and Statistical Manual V (DSM-V)* considers bipolar disorder, borderline personality disorder, major depressive disorder, schizophrenia and other forms of psychosis as forms of serious mental disorders. Defining this concept was informed by the DSM-V’s criteria. Synonyms related to the initial search term were included because of the variety of ways in which serious mental illness is defined in academia; they included but were not limited to *Mental Illness* and *Enduring Mental Illness.* A complete list of synonyms can be found in Supplementary Material 1. If an article did not explicitly state that the population being studied experienced serious mental illness, then it was removed from the analysis.

Initially, *feminism *was a search term that was required to be in the title and/or abstract of the articles to be considered for selection; however, a preliminary search found that this did not generate substantial results. The search term was broadened to include gender which generated more results; this was substantiated by the fact that feminist research places gender at the centre as an analytical category. Incorporating gender as a concept for analysis allows us to understand social reality, both concerning how knowledge is produced (Biglia and Vergés-Bosch 2016) and the effects they have on the configuration of subjectivities, and experiences, that are mediated by androcentric and heteropatriarchal constructs. A gendered analysis is critical in understanding the reproduction of power relations, which currently place certain groups (for example, women or people who do not adhere to the heteronormativity) in a situation of oppression (Harding 2004). A complete list of the synonyms used for the search can be found in Table1 (the Justification of Search Terms and list of synonyms used on the database search can be found in Supplementary Material 1).

**Table 1:** List of the synonyms

Concept A	Concept B	Concept C
Sexual*	Enduring mental illness	Feminism
Sexual*	Mental illness	Feminism
Sexual*	Mental illness	Feminist research
Sexual*	Severe mental illness	Femi*
Sexual*	Severe mental illness	Feminism
Sexual*	Severe mental illness	Feminist research
Sexual*	Severe mental illness	Gender
Sexual*	Mental disorders	Feminism
Sexual*	Mental disorders	Feminist research
Sexual*	Mental disorders	Gender
Sexual*	Mental disorders	Biopolitics
Sexual*	Mental disorders	Human rights
Sexuality	Mental health	Biopolitics
Sexuality	Mental health	Human rights
Sexual health	Mental illness	Gender

The database search generated 892 articles in total and 89 of these articles were removed for duplicates, resulting in 803 articles for further assessment. The remaining articles were screened. The title and/or abstract of these articles were assessed and were selected if they unequivocally discussed both *sexuality* and *serious mental illness*. After screening the articles, 24 articles were selected for full-text reading. Following more in-depth analysis of the 25 articles, 9 articles were later removed. In this process, the first author had done the review of the manuscripts, and the second author discussed with the first author the criteria of inclusion and exclusion. There were 16 articles selected for analysis, eight qualitative, four theoretical reflections, two quantitative, one review and one that combined quantitative and quantitative data. A description of the characteristics of the selected articles can be found in Table 2. An explanation for the removal of the 9 articles can be found in Table 3. Articles that did not exclusively mention a feminist and gendered approach in the title and/or abstract were still analysed; however, if the articles did not distinguish between gender in the results, then they were removed.

**Table 2:** Characteristics of selected articles

Reference	Country of research	Phenomenon of interest	Participants	SMI identified	Methods	Main results
Carr et al. (2015)	USA	To explore the intersections of the experience of sexual objectification and SMI among women	Women with SMI	Schizophrenia, schizoaffective disorder, bipolar disorder, major depressive disorder, severe posttraumatic stress disorder	Theoretical analysis: objectification theory	Women who experience the intersection of SMI and sexual objectification may have unique, distressing effects from the dual experiences that are particularly disenfranchising and debilitating
Cermele et al. (2001)		To explore the ways in which the DSM-IV Casebook constructs gender and race/ethnicity in depictions of individuals with mental illness	People with SMI (*n* = 145); (*n* = 55) females; (*n* = 90) males	Adult case studies in the DSM-IV Casebook	Qualitative research, thematic analysis	The DSM-IV Casebook contributes to a gendered and raced conceptualisation of mental illness, that reflect implicit definitions of normalcy
Cogan (1998)	USA	To examine what difficulties within relationship they may have needed support dealing with and how well their needs were met by community support services	Women with SMI (*n* = 25); female; aged 24–64 years	Unclear	Mixed methods; research (structured interview with scales of 1 to 10 and open-ended question), thematic analysis	Women perceived mental illness–related stigma to be an obstacle in maintaining custody of their children. They were most supported with accessing information about pregnancy, birth control and sexually transmitted diseases and least supported with their experiences of sexual abuse
Cook (2000)	USA	To explore the effects of psychiatric disability on sexual identity and behaviour	People with SMI	Severe depression, bipolar disorder, schizophrenia, personality disorder, posttraumatic stress disorder and obsessive compulsive disorder	Theoretical analysis: health consumer perspectives	There are a number of barriers that prevent sexual expression; lack of privacy in institutions, trauma, stigma, low self-esteem. Women consumers encounter special needs regarding intimacy and sexuality
Davison and Huntington (2010)	New Zealand	To gain a deeper understanding about the sexual experiences of women with SMI	Women with SMI (*n* = 8)	Unclear	Qualitative research (interviews, focus groups), thematic analysis	Women considered sexuality as a central component to their identity, but there were powerful systems that influenced their sexuality
Frieh (2019)	USA	To explore how sexual abuse and trauma impact the experience sexuality and perceive men and masculinity	Hospitalised women with SMI (*n* = 55); female	Major depression, bipolar disorder, schizophrenia, schizoaffective disorder, psychosis	Qualitative research (semi-structured interviews), abductive analysis	Trauma increases the salience of stigma and potential for retraumatisation. Labelling can perpetuate self-stigma which threatens women’s self-esteem, safety and trust in others
Hailemariam et al. (2019)	Ethiopia	To explore perspectives on marriage, divorce and family roles of women with SMI in a rural setting	Service users (*n* = 11), caregivers (*n* = 12), religious leaders (*n* = 6), health extension workers (*n* = 4), police officers, (*n* = 2), teachers (*n* = 2), government officials (*n* = 2); (*n* = 16) females, (*n* = 23) males	Psychotic disorders (i.e. schizophrenia) and major affective disorders (i.e. bipolar disorder)	Qualitative research (in-depth interviews), thematic analysis	Three themes emerged from the findings; marriage and SMI, gendered experiences of marriageability and acceptability of divorce and separation from partner with SMI
Hauck et al. (2015)	Australia	To determine associations and potential modifiable risk factors for management of sexual and reproductive health need for women attending community mental health services	Women with enduring mental illness (*n* = 220)	Anxiety, schizophrenia, bipolar mood disorder, personality disorders, eating disorders, depression	Quantitative research (survey)	Women had on average three pregnancies, majority were unplanned. One quarter who were sexually active within the past 12 months denied using contraception with 51% using less effective methods. The majority engaged in Pap smear screening
Lozano et al. (2020)	Spain	To assess the professional counselling in clinical practice based on motivational interview in women with SMI	Women with severe-moderate psychiatric disorders (*n* = 91)	Agoraphobia, anorexia, bipolar, delirious, depression, dysthymia, schizophrenia, paranoid, psychosis, personality disorder, attention deficit disorder, obsessive–compulsive disorder	Quantitative research (prospective observational cohort study)	After evidence-based counselling, 51.6% of participants changed their contraceptive method to a more effective one. This change was associated with gender violence
Lundberg et al. ( 2012)	Uganda	To explore how SMI may influence sexual risk behaviours and sexual health risks	People with SMI (*n* = 7); male (*n* = 13); female; aged 18–49 years	Schizophrenia, bipolar affective disorder or depression	Qualitative research (semi-structured interviews), content analysis	SMI and gender inequality can contribute to the shaping of sexual risk behaviours and sexual health risks
McCann et al. (2019)	Ireland	To synthesise the research on the experiences and support needs of people with SMI regarding their sexuality and intimacy in the hospital and community settings	People with SMI	SMI diagnosis from DSM-V	Systematic review	The intimate relationship and sexual experiences were synthesised into three themes: complexity of individual sexual experiences, the clinical constructs of sexuality and the family and partner involvement
Miller and Finnerty (1996)	USA	To compare sexuality, reproduction and childbearing characteristics of women with schizophrenia-spectrum disorders with those of women without SMI	Women with schizophrenia or schizoaffective disorder (*n* = 46); matched control subjects (*n* = 50)	Schizophrenia, schizoaffective disorder	Qualitative research (semi-structured interviews)	Women with schizophrenic disorders had more lifetime sexual partners, were less likely to have a current partner and were more likely to have been raped and to have engaged in prostitution. They had a higher risk of HIV infection and were less likely to have been tested. They reported wanting sex less often and rated their physical and emotional satisfaction with sex lower than control subjects. They were more likely to have lost custody of children and to report that they were unable to meet their children’s basic needs and less likely to have another caregiver helping them raise their children
Mizock and Brubaker (2021)	USA	To explore treatment experiences with mental health providers	Women with SMI (*n* = 20); female; aged 32–66 years	Depressive disorder (*n* = 10), posttraumatic stress disorder (*n* = 8), anxiety disorder (*n* = 7), bipolar disorder (*n* = 5), schizophrenia spectrum disorder (*n* = 3), schizoaffective disorder (*n* = 2), borderline personality disorder (*n* = 4), high-functioning autism (*n* = 1)	Qualitative research (semi-structured interviews), grounded theory analysis	Women with SMI perceive that they are treated differently than men with SMI by their mental health providers. This includes through diminishing dismissals, symptom misattribution, male mistrust and psychiatric insults
Perry et al. ( 2017)	USA	To analyse if theories of “soft” coercion is relevant in mental health treatment settings and to explore client experiences of choice	People with SMI (*n* = 98)	Major depressive, bipolar disorder, schizophrenia, schizoaffective disorder, psychosis	Qualitative research (in-depth interviews), thematic analysis	Identified four strategies used to influence client behaviour: coercion, enabling, education and conciliation. Women with SMI disproportionately report experiencing intense persuasion or direct use of threat of force
Swartz (2013)	South Africa	To take account of both the history and the complexity of the activity of making a psychiatric diagnosis	Women with psychiatric diagnosis	Bipolar disorder, borderline personality disorder, major depressive disorder	Theoretical analysis: reflections from a feminist perspective	The psychiatric industry is powerful: it is an accepted instrument through which human experience is categorised as either “normal” or “abnormal”. There are particular diagnostic categories that shadow patriarchal interests or represent “normal” womanhood in ways that serve patriarchal agendas
Weindhardt et al. (1999)	USA	To examine the prevalence and types of sexual coercion encountered by women with severe and persistent mental illness	Women with severe and persistent mental illness (SPMI)	Schizophrenia, schizoaffective disorder, bipolar disorder, major depressive disorder	Literature review	Sexual coercion occurs frequently in the lives of women with SPMI and that it also occurs in the context of other potential risk-conferring behaviours. Exposure to sexual violence makes women with SPMI vulnerable to a variety of sexual health complications

**Table 3:** Reasons for exclusion

Reference	Country of research	Participants	Principal reason for exclusion
Charlotte et al. (2015)	USA	Veterans with schizophrenia or bipolar disorder	Selected because discussed weight gain caused by psychotropic drugs; however, did not discuss weight gain in relation to sexuality
Hatcher et al. (2012)	USA	Women with mental disorders	Book review
Shalev et al. (2016)	Israel	People in psychiatric hospitals	Only available in Hebrew
Portocarrerro (2016)	Portugal	People with mental disorders	Only available in Portuguese
Rani et al. (2022)	N/A	Women with severe mental illness experiencing marital rape	Inaccessible
Wright (2006)	USA	People with SMI	PhD thesis
Ussher and Ussher (2011)	USA	Women with SMI	Book
Dewson et al. (2018)	England and Wales	People with mental disorders	Country-specific policy review
Welch	Canada	People in psychiatric hospitals	Country-specific policy development/review

### Eligibility criteria

#### Participants

The participants of the studies selected in this review included adult women (18 years +) with a DSMI that meets the diagnostic criteria of DSM-IV/V. The duration of time living with DSMI was not considered for the inclusion criteria. Participants also included people who work with people with DSMI and family members/partners of people with DSMI.

#### Types of studies

This research reviewed published studies that investigated the sexual experiences and sexuality of people with DSMI. Qualitative and quantitative research were both included in this review and only studies that were conducted in English were included (Table 4, Inclusion/exclusion criteria).

**Table 4:** Inclusion and exclusion criteria

Criteria	Included	Excluded
Type of study	Qualitative research; theoretical analysis, review and quantitative research	Policy reviews
Language	English	Portuguese, Hebrew, all other languages
Participants	Self-identifying women who experience SMI, people with SMI, people who work/live with people with SMI	People who do not experience SMI, people who do not work/live with people with SMI
Date	Not limited to a specified date range*	

^*^Given the continued and demonstrated impact and influence of historical practices and approaches towards women with SMI, it was considered relevant to this review to analyse data from when research initially surfaced

#### Quality assessment

Although the quality of the publications were guaranteed by their publication in indexed journals, we validated the rigour and quality of the articles according to the guidelines of the following: Critical Appraisal Skills Programme (2018) for the eight qualitative articles and for the systematic review and STROBE Checklist for quantitative studies (case control, cohort and cross-sectional studies). The article that combined qualitative and quantitative data was assessed combining the Critical Appraisal Skills programme (2018) and the Mixed Methods Appraisal Tool (MMAT) (Supplementary Material 2).

### Analysis

A thematic synthesis was used for the analysis of the selected articles (Thomas and Harden 2008) which adopts the principles of thematic analysis (Braun and Clarke 2006), to interpret empirical data for qualitative systematic reviews. Specifically, open coding of the selected articles was conducted and then a second read of the texts to assess the accuracy of the coding and to establish categories and associated subcategories. Four themes were established from eight categories (Table 5, Articles contributions to themes and categories, and Table 6, illustrations of the categories and subcategories with textual extracts from the articles analysed).

**Table 5:**
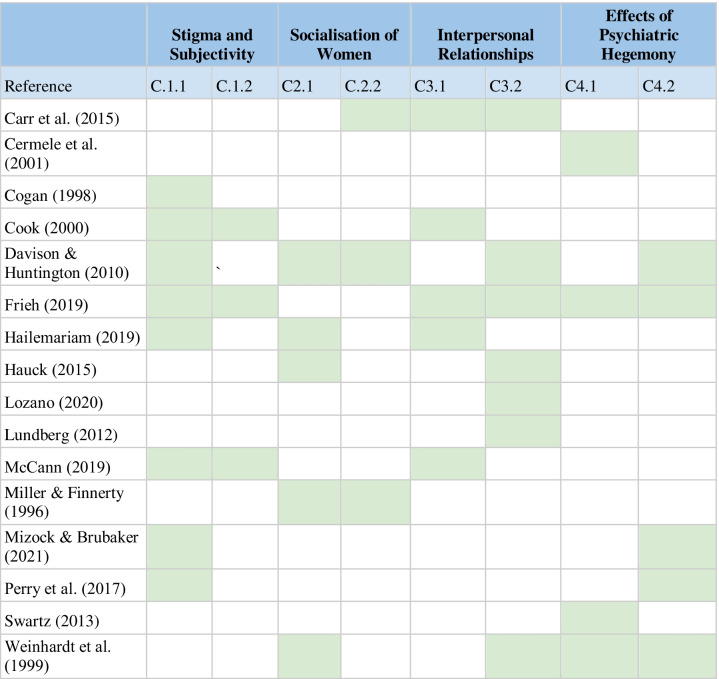
Article contributions to themes and categories

**Table 6:** Textual extracts from the articles analysed

Themes	Category	Extracts and references
Stigma and subjectivity	Category 1.1. Stigma experienced at the interpersonal and structural levels	“They advised me to use [birth control] because they didn’t think it would be healthy for me to have a baby for two reasons. One, due to my mental health, although schizophrenia has been known to skip a generation. My family members didn’t want to take the risk of me bearing a child I couldn’t take care of since I couldn’t take care of myself. Also, I had surgery on my uterus” (Perry et al. 2017. Pp- 116–117)
	Category 1.2 Internalisation of stigma	“Well, I feel really dirty and ugly and stupid, and I don’t think anybody wants me” (Davison and Huntington 2010, p.534) “Men might propose to me, but I will not be willing to marry because I am [mentally] ill. What if my illness relapses after I have got married and had children? I believe that my situation is unreliable.” (McCann et al. 2019, p.6)
Gender socialisation	Category 2.1 Gender mandates	“I didn’t realize I had learnt to put on a smiley face for everyone and I just kept closing up and closing up and deep down inside it was chewing away.” (Davison and Huntington 2010), p. 244) “…And I found that if someone was attracted to me, it was almost an obligation to have to be in a relationship or do something with them. It was like the impulse for my actions came from outside, not from me. I wasn’t tuned towards myself and my own needs or anything. I was tuned towards meeting the needs of others and survival.” (Davison and Huntington 2010, p.244)
	Category 2.2 Women objectification	“I do look down on myself and I’ve never, I mean I look at myself and I, want to throw up. I [do not] think myself as being attractive or anything and you know….” (Frieh 2020, p. 534)
Interpersonal relationships	Category 3.1 Difficulties with interpersonal effectiveness	“I’ve had a lot of experiences with men and uh, fear of being totally open and honest about some of the things I’ve struggled with for fear of rejection. You wonder if somebody goes to the bathroom and looks in the medicine cabinet, and stuff…” (Frieh 2020, 534)
	Category 3.2 Cyclical pattern of trauma	“Until someone comes that is sensitive, won’t hurt me. Wouldn’t have sex until married, would take a long time to build trust.” (Frieh 2020, 536) “He just won’t use them…Whenever I ask him [to put on a condom], the next time he’s drunk he gets mad and tells me I’m cheating on him and I’m a whore and he starts hitting me.” (Weindhart 1999, 312) “For me, when I slept on the street I was raped again. I reached a point where these street men stole all my clothes.” (Lundberg et al. 2012), p.4)
Effects of psychiatric hegemony	Category 4.1 Psychiatric diagnosis	“…for example, volatile, angry, demanding, and sexually expressive women may attract a diagnosis of borderline personality disorder, be prescribed a range of medications, and have their relational difficulties represented to their family and friends as arising from internal instability.” (Swartz 2013, p.46) “Case studies of men and women were equally likely to make reference to aspects of sexuality; however, references to men’s sexual behaviour appear in the context of diagnosis, in contrast to women as sexualized irrespective of diagnosis.” Cermele et al. 2001, p. 242)
	Category 4.2 Clinical setting	“I’ve had that thrown at me and I just keep saying…[my mental illness] it’s actually because I had abuse as a child, and no I didn’t become a lesbian because of that abuse either.” (Huntington & Davison, p. 245) “The birth control pill… I didn’t think I really needed it, but um the staff told me I had to take it… If you miss any medication while you were there, then they usually restricted you to the unit.”(Perry et al. 2018, p.114)

## Results

Based on the review, there were a total of four synthesised themes from eight categories: stigma and subjectivity, socialisation of women, interpersonal relationships and the effects of psychiatric hegemony. The dominant findings extracted from the literature discussed the processes and barriers that acted on the sexual experiences and sexuality of women with DSMI and thus, illustrated their experiences as it relates to sexuality.

### Stigma and subjectivity

Stigma has a strong impact on the daily life of women diagnosed with severe mental disorders, both in relation to the interpersonal interactions and institutional practices of oppression, and in the internalisation of stigma, which results in the deterioration of self.

#### Category 1.1. Stigma experienced at the interpersonal and structural levels

Stigma is an enduring reality for people with DSMI, and it has been noted to be as harmful as the disorder itself (Chronister et al. 2013), the effects of which range from sexual intimacy to marriageability. Stigma and stereotyping of women with DSMI can act as a form of social control over this population and can lead to cycles of victimisation and re-victimisation (Cook 2000).

Social stigma can have considerable influence on the status of romantic relationships, including in one’s desire to disclose mental health diagnosis to intimate partners and whether to initiate and engage in sexual activity (Davison and Huntington 2010; Hailemariam et al. 2019). Due to anticipated stigmatising views from the partner, a degree of trust was considered necessary for the development of intimacy and for disclosure of mental illness. Davison and Huntington (2010) and Frieh (2020) highlighted that such thought processes result from fears of rejection. People with DSMI also have more restricted social interactions due to stigma. Women with DSMI are perceived as less desirable for romantic partnership, which ultimately results in reduced perceived eligibility for marriage (Hailemariam et al. (2019).

In addition to experiencing the effects of stigma at the interpersonal level, women with DSMI are subjected to stigma at the structural and institutional levels, including beliefs that mothers with DSMI are dangerous and incapable of being parents (Cogan 1998; Perry et al. 2017; McCann et al. 2019). The effects of such stigma are presented in the findings of Cogan (1998) and McCann et al. (2019) which demonstrate the inequitable reality of mothers with DSMI, who have to prove their parenting skills and unjustifiably fight for child custody (Cogan 1998; McCann et al. 2019). Such reports of being delegitimised was also experienced when women with DSMI perceived that their reports of abuse were neglected because of their healthcare providers stigma towards mental illness diagnosis (Mizock and Brubaker 2021). These attitudes are compounded by the lack of sexual, reproductive and mental health training that medical professionals receive (Perry et al. 2017).

#### Category 1.2 Internalisation of stigma

Internalisation of stigma was recurrent in the lives of women with DSMI. This internalisation was in part due to this population’s vulnerable societal position, which includes being stigmatised because of their mental health diagnosis, and the ascribed societal expectations associated with being a woman (Frieh 2019).

As demonstrated in the findings that follow, self-stigma pervades many facets of the lived experience of women with DSMI. Internalised stigma can result in negative perceptions of self, including lowered self-esteem, self-confidence and self-worth. Frieh (2019) points out that women with DSMI perceived themselves as being unattractive and inadequate partners. The effects of this can create difficulties in initiating and maintaining intimacy (Cook 2000; Frieh 2019; McCann et al. 2019). Internalisation of stigma had been noted to affect the subjective experience, with participants having indicated perceiving a sense of societal disapproval of their sexuality as it relates to their DSMI, which led to sexual repression, and dissatisfaction of sexual activity and overall sexuality of women with DSMI.

### Gender socialisation

The socialisation of women, gender biases and powerful social structures entrenched in gender norms lead to barriers that influence the fulfilment of sexuality and sexual expression. We present the findings related to the prescriptions that heteropatriarchal discourses make about women’s roles in society and the specific effects on beliefs associated with motherhood and relationships for DSMI women, as well as their effects on the reification of the body as an object of desire of the other.

#### Category 2.1 Gender mandates

The pressure to conform to societal expectations can have a significant impact on the experience of sexuality. Such conformity can result in suppression of genuine feelings, which can create conflict between one’s desire, and what one is supposed to do and feel according to social mandates (Weinhardt et al. 1999; Hailemariam et al. 2019). In the review, it was found that participants experienced pressure to appease others and to maintain the roles of passive and submissive. The obligation to meet others’ needs and remain silent was found to have occurred in multiple circumstances within participants’ lives including in their role in negotiating sex and marriage (Davison and Huntington 2010; Hailemariam et al. 2019).

Women’s status and value in heteropatriarchal societies are frequently related to their ability to be good and diligent partners and mothers. As highlighted by Hailemariam et al. (2019), firstly, marriage eligibility for women with DSMI was associated with their ability to fulfil prescribed gender roles. Secondly, women with DSMI face more challenges with parenting than women without DSMI, as stigma permeates and challenges the role of mothers and places them in a situation of greater relational and material vulnerability. More specifically, women with schizophrenia spectrum disorders were more likely to be raising a child without support from another person, and they were more likely to have someone else raising their child altogether (Miller and Finnerty (1996).

Gender-specific societal expectations leave women with DSMI with feelings of diminished autonomy and worsened perception of self, which prevents women’s full control over safe sexual practices and their sexuality (Davison and Huntington 2010; Hauck et al. 2015).

#### Category 2.2 Women objectification

The recurrent objectification and subjugation of women’s bodies, through insidious forms including catcalling, as well as more explicit forms of trauma such as sexual abuse affect the practical and subjective sexual experiences of women with DSMI. Sexual objectification, of which women with DSMI are more vulnerable, can lead to intense scrutiny of women’s bodies by themselves and can cause problems with self-image and results in worsened mental health outcomes (Carr et al. 2015). The interpersonal effects of such objectification include women with DSMI feeling stigmatised and “othered” due to their body size, particularly given that weight gain is a side effect of psychotropic medication (Davison and Huntington 2010).

The internalisation of fat-phobic discourses and body shaming can lead to self-defence mechanisms including dissociation from one’s body and hypervigilance of one’s surroundings. This can have pervasive effects on an individual’s sense of self, their mental health and their social lives (Miller and Finnerty 1996; Davison and Huntington 2010). The inability to connect with others and social isolation are further exacerbated by the trauma which accompanies different forms of sexual objectification.

### Interpersonal relationships

Interpersonal relationships are complex and difficult to navigate but are an important element of sexual experiences and in the fulfilment of sexuality. Like every other population, women with DSMI hold desire for human connection and expressed needs for intimacy (McCann et al. 2019).

#### Category 3.1 Difficulties with interpersonal effectiveness

Establishing relationships can be challenging for women with DSMI for a variety of reasons (Cook 2000; Carr et al. 2015). A stable perception of self and the ability to be vulnerable and open with others are considered necessary for engaging in sexual activity and intimacy; however, women with DSMI can frequently experience diminished levels of self-confidence and self-worth (Cook 2000).

Symptoms and manifestations of mental disorders (i.e. experience of paranoia, averted eye contact) might be perceived as anti-social and could result in social withdrawal and isolation, making it difficult to establish relationships (Cook 2000). The reviewed studies highlight that societal perceptions of people with DSMI including perceptions of being dangerous, “other” and other stigmatising beliefs created difficulties when establishing relationships for women with DSMI (Cook 2000; Hailemariam et al. 2019). Internalised stigma further exacerbated existing challenges to establishing relationships and intimacy and acted as a self-fulfilling prophecy by perpetuating the sense of “otherness” and mistrust (Cook 2000; Frieh 2019; Hailemariam et al. 2019).

Difficulties maintaining intimate relationships were also found to be barriers in the fulfilment of sexuality. Studies found that living with DSMI can negatively affect intimate and romantic relationships by placing pressure (Hailemariam et al. 2019; McCann et al. 2019). Partners of women with DSMI discussed the strain that DSMI had on their relationship, citing the perceived need to monitor and “stay on top” of their partner’s illness (McCann et al. 2019). Some women with DSMI accepted their partners’ decisions to have sex with other people, particularly during an acute mental health episode (Lundberg et al. 2012).

#### Category 3.2 Cyclical pattern of trauma

Trauma and stigma are factors that severely influence the desire and ability to engage in intimate relationships for women with SMI. Women with DSMI disproportionately experience abuse and sexual exploitation by partners and strangers when compared to the general population (Weinhardt et al. 1999; Lundberg et al. 2012). Economic vulnerabilities and dependence on psychotropic medication created a reliance on the need to remain in or engage in exploitative or coercive situations (such as partner abuse, sexual exploitation by clinician or sex work) (Lundberg et al. 2012). Such economic reliance on abusive partners inhibits a woman’s ability to employ their desired contraception and to establish autonomy (Lundberg et al. 2012; Carr et al. 2015). However, upon receiving contraceptive counselling, women with DSMI who had previously experienced gender violence were more likely to opt for more effective contraceptive methods than women who had not previously experienced gender violence (Lozano et al. 2020). Such experiences of abuse were noted to affect self-esteem and a woman’s ability to trust in a relationship. These experiences result in self-narratives of being “broken” and undesirable and were found to discourage study participants from pursuing relationships (Frieh 2020).

Women with DSMI can be vulnerable to a cycle of stigma and trauma, wherein increased stigma is experienced, due to diagnosis of mental illness, which leads to increased likelihood of sexual assault and coercion. This outcome leads to more trauma and consequently worsened perception of self (Davison and Huntington, 2015). These exposures create a vulnerability to economic insecurity and establish ground for the cycle to continue in an omnipresent manner.

### Effects of psychiatric hegemony

Psychiatric hegemony is the power dynamic used by the institution of psychiatry to exert control over individuals to sustain the values of the dominant society (Stragalinos 2018). Women with DSMI faced multi-faceted challenges including being subjected to dominant powers within psychiatry, particularly as it relates to their sexual orientation, race, gender and mental health diagnoses. From the analysis of the studies carried out, it can be noted that the psychiatric institution might play an important role in the repression of the sexuality of women with DSMI and also might function to ensure that the women conform to dominant hegemonic principles and systems. As a result of the specific position in society of women with DSMI, their relationships to these dominant systems were found to have an effect on their sexual experiences and fulfilment of their sexuality.

#### Category 4.1 Psychiatric diagnosis

Some authors highlight that psychiatric diagnoses maintain white heteropatriarchal norms through the objectification of women’s bodies and through the medicalisation of the psychological experience (Weinhardt et al. 1999; Cermele et al. 2001). Women with DSMI are already at increased vulnerability of medical intervention and psychiatric control, and the medicalisation of femininity further increases this exposure and risk (Frieh 2019). Such medicalisation bolsters societal constructs that women are fragile and in need of protection and thus further disadvantages them, overtly in the healthcare system and covertly in their perceptions of self (Frieh 2019).

Women with DSMI, in comparison to men with DSMI, had their ordinary emotional reactions pathologised during diagnosis and treatment, in part due to healthcare professionals attributing positive emotional wellbeing to men and not women (Weinhardt et al. 1999). Such discrimination can be found in the prevalence of borderline personality disorder diagnoses among women compared to men. Wherein women who express anger or volatility may receive a diagnosis of borderline personality disorder which may result in greater reliance on psychiatric institutions and psychotropic drugs, as well as difficulties with relationships (Swartz 2013). Responses to femininity are reflected in the way in which the psychiatric institution defines psychopathologies, which results in women being hypersexualised and overdiagnosed and therefore increases their vulnerability to be controlled (Cermele et al. 2001; Swartz 2013). This is exemplified in the Diagnostic and Statistical Manual Casebook, where aspects of women’s sexuality were referenced regardless of their diagnosis, while men’s sexual behaviours were referenced solely as it related to a diagnoses (Cermele et al. 2001).

Racialised people are consistently overdiagnosed with serious mental illness (Cermele et al. 2001). In particular, racialised women with DSMI are twice as targeted in the diagnostic process as white women with DSMI. The referencing of race and people of colour in the Diagnostic and Statistical Manual Casebook occurs without any context as to how and/or if it relates to diagnosis (Cermele et al. 2001). This creates the impression that race is made noteworthy but only when it applies to people of colour and sustains the hegemonic belief that whiteness is the “norm”.

#### Category 4.2 Clinical setting

The clinical setting acts as a barrier for sexuality and sexual expression of women with DSMI. Psychiatric hospitalisations are frequent in the lives of women with DSMI; however, hospital conditions, including a lack of safety and privacy, do not facilitate effective treatment or the establishment of healthy relationships. In the studies of this review, gendered power differences were noted in clinic settings. Gender dynamics between women with DSMI and male providers were noted as having gone unaddressed (Mizock and Brubaker 2021)). The result of such dynamics and gender relations led women with DSMI to question the integrity of their psychiatric diagnosis and treatment by male clinicians (Mizock and Brubaker 2021). Coercive strategies within the healthcare system specifically towards women with DSMI further diminished their autonomy and decision-making capacity over their bodies (Perry et al. 2017). These strategies were implemented through the use of threat or force (Perry et al. 2017). The studies of this review found that psychiatric hospitals were also noted as a setting for sexual violence (Frieh 2019). Specifically, cases are reported in which women with DSMI were not able to implement boundaries against sexual abuse by mental health providers, because of their concern or experiences with being discredited due to their gender and mental health diagnosis (Weinhardt et al. 1999).

The hostile environment is exacerbated for women with DSMI who experience homophobia. The pathologisation of sexuality and the stigma associated with being a lesbian prompted some participants in the studies to hide their sexual orientation and culminated in what was considered a “double coming out” and “double stigma” (Davison and Huntington 2010).

## Discussion

The findings suggest that stigma acts on women with DSMI at the micro-social level through interactional processes and self-stigma and also at a structural level, through institutional practices of sexual rights vulneration. Link and Phelan (2001) note that structural stigma alters the scaffolding around a person, which leads to exposure of inopportune and unpredicted conditions. Consider the articles of this review that found that women with DSMI were more likely to experience sexual objectification and sexual abuse than women without DSMI. Women with DSMI often encounter economic trouble (in part because of the stigma surrounding mental illness), subsequently leading to higher rates of sexual objectification and abuse (Lundberg et al. 2012). While stigma might not have been necessarily discernible at the moment of trauma, it certainly plays a critical role in creating vulnerability to trauma for women with DSMI. Stigma can be subtle yet consequential for women with DSMI and was found to pervade many of the sexual experiences of women with DSMI. This demonstrates that women with DSMI are disadvantaged at multiple levels (i.e. interactional, structural) by the negative effects of psychosocial processes as stigma. These processes operate to oppress women with DSMI, particularly their sexual experiences and sexuality.

Processes of stigma and gender oppression not only occur at the conceptual, cognitive level; but they materialise into real consequences within the social lives of women with DSMI, which then act to transform their subjective experiences. A subject is shaped entirely by social and cultural processes in a given society, and so, an individual is not born a subject, but rather becomes one (Sheikh 2017). Gender is a social construct that acts on subjectivity. From the time an individual is born, there are processes that inform them of their gender and influence their subjective experience. In a patriarchal society, a female becomes a woman through gendered processes and continually learns of their role in relation to men. As noted by Beauvoir, there is a pattern of social acceptance that considers men as subjects and women as their objects (Gill and Pires 2019). This affects the ongoing conditions of a woman’s life, including their opportunities and risks. Consider the findings that described women with DSMI as needing to maintain a happy and passive demeanour, and feeling required to silence their needs (Cook 2000). These experiences were informed by internalised stereotypes and reflect dichotomous power relations of women being objects (and men being subjects).

Langman (1998) suggests that the insidiousness of transforming subjectivity was the “magic” behind reproduction of hegemonic power and ideologies, because it essentially sustains the subject’s own subjugation of self. As Esteban and Távora (2008) explain, the construction of women’s subjectivity serves to reproduce oppression and to maintain the prevailing Western heteropatriarchal model. Where male sexual desire configures women’s subjectivity, from the perspective of being looked at and desired: “Femininity constructs us with an identity, prevalently centred on a being to be perceived, to be looked at, has the effect of placing us in a state of permanent bodily insecurity and, simultaneously, of symbolic alienation” (Dio Bleichmar 2000, p. 188 in Esteban and Távora 2008, p. 63–64 [own translation]). This*prevalence of being to be looked at and desired*comes into conflict with the stigma of DSMI. Consider the findings that reported women with DSMI as having internalised stereotypes related to mental health stigma that people with DSMI are sexually unattractive. This language became a means for self-identification. In some cases, this led to participants choosing to date only people with DSMI or led to sexual repression (Cook 2000; Lundberg et al. 2012). In this example, it is clear how stigma and socially constructed dimensions of value act to impose normative criteria of being (in this case, as it relates to attractiveness and unattractiveness) to transform and control the subjective experience of women with DSMI.

Lastly, subjectivity is transformed in the way that the effects of normative discourses and stigma occupy the thoughts of women with DSMI. This was materialised overtly in their experiences, through noted hypervigilance and body monitoring (Carr et al. 2015), and more covertly, in the way in which women with DSMI needed to constantly prove their capacity to parent (Hailemariam et al. 2019; McCann et al. 2019; Perry et al. 2017). This aspect shows how the stigma experienced puts into question another elements that, within Western heteropatriarchal societies, serve to configure female subjectivity, “the power of affects” as a value to help, to sustain and to grow others (Mabel Burin 2008), where motherhood is an essential component (Esteban and and Távora 2008). These elements of subjectivity create less opportunity for women with DSMI to self-advocate and diminish their agency, which ultimately perpetuates the cycle of their subordination within the heteropatriarchal system.

Most of the articles in this review focused on gender and diagnosis of severe mental disorders as essential elements of problematisation in the production of subjectivity and interactional dynamics. We have also identified studies, which complete their analysis by introducing other non-hegemonic categories that act on the individual. This allows us to delve into how the processes of stigmatisation and its effects manifest and thus interact with other categories of oppression such as sexual orientation racialisation and the non-normative body (Davison and Huntington 2010; Cermele et al. 2001; Cook 2000). Such studies highlight the need to adopt an intersectional approach in the exploration of the effects of the heteropatriarchy and medical hegemony on subjectivity and in the reproduction of oppressive systems. These acquire multiple expressions and intensities concerning other non-hegemonic social identities.

The studies allow us to problematise the current situation of violation of the sexual and reproductive rights of women diagnosed with severe mental disorders. They highlight how the heteropatriarchal system and the stigma associated with the diagnosis act together to produce an increased risk of experiencing structural conditions of vulnerability and fragile and inequitable relationships. Similarly, to their effects on the subjective experience, women with DSMI are marked by the tensions of gender mandates and the devaluation of identity caused by the stigma of mental disorders.

However, in everyday practice, there are various experiences of women’s movements that struggle to break with such stigmas and gender mandates; including the militancy for the rights of people with disabilities and women diagnosed with SMI. An example of this is in Spain, where associations composed of such populations, their family members and non-governmental and professional organisations advocate to make visible the experience of sexual and reproductive rights violations (Santoro Lamelas et al. 2022). This advocacy and support takes the form of mutual aid groups as well as the establishment of strategic alliances with public, academic and community institutions in order to problematise institutional practices and promote proposals for women empowerment (Federació Salut Mental Catalunya 2022). As well, as a result of the collective claims and in agreement with the Convention of the Rights of Persons with Disabilities (2006), Spain recently approved of the Organic Law 2/2020, which aims to eradicate forced sterilisations of people with disabilities, including women with SMI (Prados García 2021). This law represents a step forward in rethinking the exercise of sexual and reproductive rights of women with SMI.

It is necessary to place women with a diagnosis of SMI at the centre of to study designs and political action to recognise and to further facilitate their agency over the exercise of their sexual and reproductive rights. It is also necessary to address the potential of community action as a resource that allows for a broader perspective and moves away from the discourse centred on the medical diagnosis and the structural limitations associated with it. This can enable the strengthening of spaces for support, companionship and empowerment as it relates to the exercise of the sexual and reproductive rights.

## Conclusion

Women with DSMI experienced barriers to the fulfilment of sexuality that are unique to the social position that they occupy. The research found that the sexual experiences and sexuality of women with DSMI are often dictated by stigma, their socialisation as women, their interpersonal relationships and the hegemonic psychiatric institution with which they interact. These barriers interconnected to affect the sexual experiences of women with DSMI at the subjective, interpersonal and structural levels.

These mechanisms work to silence and oppress the sexuality of women with DSMI by creating a culture of using dichotomous thinking and stigmatising language and behaviours. These processes also act on the subjective experience of women with DSMI, which results in diminished self-worth, unsatisfying social interactions and social withdrawal. Through these processes, hegemonic systems of power are reproduced, which perpetuates the invisibility, neglect and control of sexuality and sexual experiences of women with DSMI by dominant systems.

## Supplementary Information

Below is the link to the electronic supplementary material.

Supplementary file1 (DOCX 28 KB)

Supplementary file2 (DOCX 40 KB)
