# Supplementary material for: Feminist contributions on sexual experiences of women with serious mental illness: a literature review
Source: Arch Womens Ment Health. 2022 Aug 22;25(5):853–70. doi: 10.1007/s00737-022-01258-0 (PMC9492617; doi:10.1007/s00737-022-01258-0)
Supplement: Supplementary file 1 — Supplementary file1 (DOCX 28 KB) [file 737_2022_1258_MOESM1_ESM.docx]

***Supplementary Material 1:*** *Justification of Search Terms and list of synonyms used on the database search.*

**Concept A** involved various forms of the word ‘sexual-‘ for comprehensiveness, but ‘sexuality’ was the most commonly searched term for Concept A. According to the WHO sexuality is vast and encompasses gender identity, intimacy and reproduction among many other facets of life (1). The WHO states that sexual health is “not operational without a broad consideration of sexuality” (1), in other words, defining and understanding sexuality precedes defining and understanding sexual health. By this logic, using the search term ‘sexuality’ was also encompassing the above noted-terms and this is why it was selected as a search term.

**Concept B** sought to identify study participants or study populations living with SMI. The DSM-5 considers schizophrenia-spectrum disorders, severe bipolar disorder, major depressive disorder, borderline personality disorder and other forms of psychosis as forms of serious mental disorders. Therefore, the process of defining Concept B was informed by the DSM-5’s criteria of SMI, or serious mental disorders. Synonyms related to the search of Concept B can be found in Table 1, other search terms include ‘Mental Illness’ and ‘Enduring Mental Illness’. Not all enduring mental illnesses are considered serious mental illnesses; however, for comprehensiveness this term was included in the search strategy. This was done because of the variation of ways in which serious mental disorder is defined in academia.

**Concept C** was used to correspond to the focus on women and feminist research, for this reason, terms such as “gender” and “feminist research” among others, were used. Other search terms were employed, including “biopolitics” and “human rights”. Prior to conducting the database search, preliminary research was conducted that revealed that there were distinguishable gendered experiences as it relates to biology, politics and human rights. This was the rationale for substituting these terms, as a means of deepening the understanding of this social position.

## **List of the Synonyms**

| **Concept A** | **Concept B** | **Concept C** |
| --- | --- | --- |
| Sexual* | Enduring Mental Illness | Feminism |
| Sexual* | Mental Illness | Feminism |
| Sexual* | Mental Illness | Feminist Research |
| Sexual* | Severe Mental Illness | Femi* |
| Sexual* | Severe Mental Illness | Feminism |
| Sexual* | Severe Mental Illness | Feminist Research |
| Sexual* | Severe Mental Illness | Gender |
| Sexual* | Mental Disorders | Feminism |
| Sexual* | Mental Disorders | Feminist Research |
| Sexual* | Mental Disorders | Gender |
| Sexual* | Mental Disorders | Biopolitics |
| Sexual* | Mental Disorders | Human Rights |
| Sexuality | Mental Health | Biopolitics |
| Sexuality | Mental Health | Human Rights |
| Sexual Health | Mental Illness | Gender |

## 
