# Supplementary material for: Feminist contributions on sexual experiences of women with serious mental illness: a literature review
Source: Arch Womens Ment Health. 2022 Aug 22;25(5):853–70. doi: 10.1007/s00737-022-01258-0 (PMC9492617; doi:10.1007/s00737-022-01258-0)
Supplement: Supplementary file 2 — Supplementary file2 (DOCX 40 KB) [file 737_2022_1258_MOESM2_ESM.docx]

| Qualitative studies |  |  |  |  |  |  |  |  |  |  |  |  |
| --- | --- | --- | --- | --- | --- | --- | --- | --- | --- | --- | --- | --- |
| Title | Authors | Ckecklist | Q1 | Q2 | Q3 | Q4 | Q5 | Q6 | Q7 | Q8 | Q9 | Q10 |
| Defining Normal: Constructions of Race and Gender in the DSM-IV Casebook. | Cermele et al. (2001) | CASP. Qualitative studies. | Y | I | Y | NA | NA | NA | NA | I | Y | V |
| “Out of sight”: Sexuality and women with enduring mental illness. | Davison, J., & Huntington, A. (2010) | CASP. Qualitative studies. | Y | Y | Y | I | Y | Y | Y | Y | Y | V |
| Stigma, trauma and sexuality: the experiences of women hospitalised with serious mental illness. | Frieh, E.C. (2019) | CASP. Qualitative studies. | Y | Y | Y | Y | Y | I | Y | Y | Y | V |
| “He can send her to her parents”: The interaction between marriageability, gender and serious mental illness in rural Ethiopia. | Hailemariam et al. (2019) | CASP. Qualitative studies. | Y | Y | Y | Y | Y | N | Y | Y | Y | V |
| Sexual Risk Behaviours and Sexual Abuse in Persons with Severe Mental Illness in Uganda: A Qualitative Study. | Lundberg et al. (2012) | CASP. Qualitative studies. | Y | Y | Y | Y | Y | N | Y | Y | Y | V |
| Treatment experiences with gender and discrimination among women with serious mental illness. | Mizock & Brubaker (2021) | CASP. Qualitative studies. | Y | Y | Y | Y | Y | N | Y | Y | Y | V |
| Therapeutic Social Control of People with Serious Mental Illness: An Empirical Verification and Extension of Theory. | Perry et al. (2017) | CASP. Qualitative studies. | Y | Y | Y | Y | Y | N | Y | Y | Y | V |

**Supplementary material 2: Quality Evaluation of the selected papers.**

Abbreviations: Yes: Y; No: N; Not applicable: NA; I: More information on these aspects is desirable; V: The research explains its value adequately.

| Quantitative studies |  |  |  |  |  |  |  |  |  |  |  |  |  |  |  |  |  |  |  |  |  |  |  |  |
| --- | --- | --- | --- | --- | --- | --- | --- | --- | --- | --- | --- | --- | --- | --- | --- | --- | --- | --- | --- | --- | --- | --- | --- | --- |
| Title | Authors | Checklist | Q1 | Q2 | Q3 | Q4 | Q5 | Q6 | Q7 | Q8 | Q9 | Q10 | Q11 | Q12 | Q13 | Q14 | Q15 | Q16 | Q17 | Q18 | Q19 | Q20 | Q21 | Q22 |
| Sexuality, pregnancy, and childrearing among women with schizophrenia-spectrum disorders. | Miller & Finnerty (1996) | STROBE Statement. Case-control studies | Y | Y | I | Y | Y | Y | Y | Y | I | Y | Y | Y | Y | Y | Y | Y | NA | Y | N | N | N | Y |
| Professional counseling in women with serious mental illness: achieving a shift toward a more effective contraceptive method | Lozano et al. (2020) | STROBE Statement. Observational studies | Y | Y | I | Y | Y | Y | Y | Y | Y | Y | Y | Y | Y | Y | Y | Y | Y | Y | Y | Y | Y | N |
| Sexual and Reproductive Health Trends Among Women With Enduring Mental Illness: A Survey of Western Australian Community Mental Health Services | Hauck et al. (2015) | STROBE Statement. Cross-sectional studies | Y | Y | I | Y | Y | Y | Y | Y | I | Y | Y | Y | Y | Y | Y | Y | NA | Y | Y | Y | Y | Y |

Abbreviations: Yes: Y; No: N; Not applicable: NA; I: More information on these aspects is desirable.

| Others |  |  |  |  |  |  |  |  |  |  |  |  |  |  |  |  |  |
| --- | --- | --- | --- | --- | --- | --- | --- | --- | --- | --- | --- | --- | --- | --- | --- | --- | --- |
| Title | Authors | Checklist | Q1 | Q2 | Q3 | Q4 | Q5 | Q6 | Q7 | Q8 | Q9 | Q10 |  |  |  |  |  |
| Sexuality and intimacy among people with serious mental illness. | McCann et al. (2019) | CASP. Systematic Review | Y | Y | Y | Y | Y | Y | Y | Y | Y | NA |  |  |  |  |  |
| Title | Authors | Checklist | Q1.1 | Q1.2 | Q1.3 | Q1.4 | Q1.5 | Q4.1 | Q4.2 | Q4.3 | Q4.4 | Q4.5 | Q5.1 | Q5.2 | Q5.3 | Q5.4 | Q.5.5 |
| The consumer as expert: Women with serious mental illness and their relationship-based needs. Cogan, J.C. (1998). | Cogan, J.C. (1998) | Mixed Methods appraisal tool (MMAT) (2018) | Y | Y | Y | Y | I | Y | I | Y | Y | Y | Y | Y | Y | N | I |
| Feminism and psychiatric diagnosis: Reflections of a feminist practitioner. | Swartz, S. (2013) | NA | Theoretical paper. Reflection from cases. | | | | | | | | | | | | | | |
| Sexual coercion among women living with a severe and persistent mental illness. | Weinhardt et al. (1999) | NA | Theoretical paper and reflection from research | | | | | | | | | | | | | | |
| Sexuality and people with psychiatric disabilities. | Cook, J.A. (2000) | NA | Theoretical paper and reflection from research. | | | | | | | | | | | | | | |
| Women and the Experience of Serious Mental Illness and Sexual Objectification: Multicultural Feminist Theoretical Frameworks and Therapy Recommendations. | Carr et al. (2015) | NA | Theoretical paper | | | | | | | | | | | | | | |

Abbreviations: Yes: Y; No: N; Not applicable: NA; I: More information on these aspects is desirable.
